# Design principles for fully online flipped learning in health professions education: a systematic review of research during the COVID-19 pandemic

**DOI:** 10.1186/s12909-022-03782-0

**Published:** 2022-10-13

**Authors:** Chung Kwan Lo, Khe Foon Hew

**Affiliations:** 1 Department of Mathematics and Information Technology, The Education University of Hong Kong, Hong Kong SAR, China; 2 Faculty of Education, The University of Hong Kong, Hong Kong SAR, China

**Keywords:** COVID-19, Flipped learning, Flipped classroom, Inverted classroom, Systematic review

## Abstract

**Background:**

During the COVID-19 pandemic, some instructors transitioned their courses into a fully online environment by adopting flipped learning. In this context, this review examined the challenges to fully online flipped learning and identified useful course-design elements for practicing this instructional approach in health professions education.

**Methods:**

We followed the Preferred Reporting Items for Systematic Reviews and Meta-Analysis (PRISMA) statement for selecting relevant articles. Thirty-three empirical studies (with 32 unique interventions) published between 2020 and 2021 (i.e., the first 2 years of the pandemic) were selected for analysis.

**Results:**

When the instructors in the reviewed studies designed and implemented their online flipped courses, numerous challenges emerged, which could be broadly categorized into student-related challenges (e.g., unfamiliarity with online flipped learning; *N* = 5), faculty challenges (e.g., increased workload; *N* = 8), and operational challenges (e.g., students’ technical problems; *N* = 9). Nevertheless, we identified various useful elements for online flipped learning practice and organized them based on the following components of the Revised Community of Inquiry (RCoI) framework: cognitive presence (e.g., application of knowledge/skills; *N* = 12), social presence (e.g., peer interaction; *N* = 11), teaching presence (e.g., instructors’ real-time demonstration/facilitation; *N* = 17), and learner presence (e.g., care and emotional support; *N* = 4).

**Conclusions:**

Based on the findings from the review and the RCoI framework, we developed nine principles for the effective practice of online flipped learning. These principles appear crucial for sustaining quality health professions education in a fully online flipped learning environment.

**Supplementary Information:**

The online version contains supplementary material available at 10.1186/s12909-022-03782-0.

## Background

The COVID-19 pandemic has triggered a worldwide crisis in the health professions, as it has not only increased pressure on healthcare systems but has also profoundly impacted health professions education. Owing to measures adopted to control the pandemic, which have included social distancing policies, universities and training sites across numerous countries were closed to onsite learning for extended periods. For example, the guidelines of the Association of American Medical Colleges recommended that clinical rotations for medical students be paused in medical schools in the United States [1*]. In China, colleges and universities were required to deliver courses online in early 2020 [2*]. All teaching and learning activities were conducted in a fully online environment via video conferencing platforms (e.g., Zoom).

During the pandemic, some instructors adopted flipped learning instead of traditional lecture-based instruction remotely online, as previous studies showed that flipped learning could enhance students’ responsibility, sense of belonging to a group, interactions, and learning motivation [3*]. In this context, what were their experiences of practicing fully online flipped learning? How can we improve the design and implementation of this instructional approach? This review aims to address these very questions.

We first seek to understand the challenges to online flipped learning in the health professions that have been reported across studies. We then identify the elements that have been useful to instructors and students for course design and implementation. Our overarching goal is to synthesize the previous research in order to establish a set of principles for enhancing the practice of online flipped learning in the health professions. The development of these principles will allow us to use this instructional approach not only as a fallback or contingency plan in the face of future pandemics but also as an alternative approach to supplement regular teaching and learning activities. The following are the research questions (RQ1 and RQ2) that guided this review.

- RQ1: What are the challenges to online flipped courses during the pandemic?
- RQ2: What are the useful elements of the design and implementation of online flipped courses?

## Conceptual background

Although teaching and learning activities were shifted online, these online activities were not necessarily delivered using flipped learning. Therefore, we first clarify the definition of this instructional approach. After that, we develop our frameworks for analyzing its challenges and course-design elements.

### Definition of online flipped learning

As described by Oudbier et al. [4], flipped learning reverses the instructional sequence of traditional lecturing, as students use digital learning tools and resources (e.g., instructional videos) to acquire knowledge on a topic before class. More class time can thus be spent on interactive learning activities, such as collaborative problem-solving and group discussions. This echoes the call for a shift in health professions education from the traditional lecture-based approach toward an instructional approach that encourages active learning and higher-order thinking [5]. Before the pandemic, Hew and Lo [6] analyzed 28 studies comparing the flipped and traditional approaches in health professions education. Their meta-analysis indicated that flipped learning could improve student achievement with a small but significant effect size. Furthermore, student perceptions of flipped learning have generally been found to be positive [5, 6].

During the pandemic, face-to-face class activities have had to be conducted online via video conferencing platforms such as Zoom (e.g., [1*, 7*, 8*]). Under this restriction, the definition of flipped learning offered by the Flipped Learning Network [9] is still relevant because it does not impose constraints on the instructional media used for either pre-class or in-class learning. Instead, the definition emphasizes the instructional sequence of first using the individual learning space for direct instruction (pre-class) and then subsequently using the group learning space for interactive knowledge application activities (in-class). As this definition of flipped learning is also suitable for contexts in which the group learning space is virtual, we adopted the definition without imposing additional constraints on the instructional media and activities used in online flipped courses.

### Analytical Framework for challenges to online flipped learning

Despite the potential advantages of flipped learning, there are challenges to its practical application. Before the pandemic, Betihavas et al. [10] classified the challenges to flipped learning that were identified across studies in nurse education into three categories: (a) student-related challenges, (b) faculty challenges, and (c) operational challenges. To verify the relevance of these categories for online flipped learning, we conducted a preliminary exploration of relevant studies published in “Academic Radiology” [1*, 11*] and “BMC Medical Education” [2*, 8*], both of which are leading journals in health professions education. Our initial findings generally conformed with the above categorization. For example, in-person interactions and hands-on practices have been found to be unfeasible in a fully online environment [1*, 11*], which could be classified as a kind of operational challenges. Therefore, we adopted the framework of Betihavas et al. [10] for analyzing the challenges to online flipped learning across studies.

### Theoretical framework for course-design elements in flipped learning

MacLeod et al. [12] argued that theoretical frameworks can help medical educators improve their instructional practices. A highly cited article by Kim et al. [13] formulated nine design principles for flipped learning based on their experiences with three undergraduate flipped courses (engineering, social studies, and humanities) before the pandemic. These design principles were theoretically grounded on the Revised Community of Inquiry (RCoI) framework [14–17]. The RCoI framework theorizes that the following four components contribute to a successful learning environment.

- Cognitive presence: the extent to which students are able to construct meaning and understanding in a CoI, in which they move deliberately from understanding to exploration, integration, and ultimately to application.
- Social presence: the ability of students to project themselves socially through the communication medium in use. Effective communication, open communication, and group cohesion are the three main aspects of social presence.
- Teaching presence: the planning that goes into the design, facilitation, and direct instruction in learning environments.
- Learner presence: the self- and co-regulatory strategies that students use to marshal their thoughts, emotions, motivation, behavior, and learning strategies.

Table 1 summarizes the nine design principles formulated by Kim et al. [13]. Each principle supports one of the abovementioned types of presence in a flipped learning environment. However, Kim et al. [13] acknowledged that “the nine principles might not always work in every classroom instance” (p. 46) because they were formulated based solely on the context of an urban university in the United States. Therefore, this review aims to refine and contextualize the existing principles to address the current needs in health professions education.

**Table 1:** Design principles for flipped learning formulated by Kim et al. [13] by type of presence

Type of presence	Design principles
Cognitive presence	• Provide an opportunity for students to gain first exposure prior to class • Provide clear connections between in-class and out-of-class activities • Provide clearly defined and well-structured guidance
Social presence	• Provide facilitation for building a learning community • Provide technologies that are familiar and easy to access
Teaching presence	• Provide an incentive for students to prepare for class • Provide a mechanism to assess student understanding • Provide prompt/adaptive feedback on individual or group work
Learner presence	• Provide enough time for students to carry out assignments

## Methods

### Search strategies

We followed the Preferred Reporting Items for Systematic Reviews and Meta-Analysis (PRISMA) statement [18] when selecting relevant studies. We searched the following 11 electronic databases: (1) Academic Search Ultimate, (2) APA PsycInfo, (3) British Education Index, (4) CINAHL Complete, (5) Education Research Complete, (6) EMBASE, (7) ERIC, (8) MEDLINE, (9) PubMed, (10) Scopus, and (11) Web of Science. The search string, including the relevant keywords and Boolean operators, was as follows: (flip* OR invert*) AND (class* OR learn* OR instruction* OR course*) AND (COVID* OR coronavirus OR pandemic). The asterisk was used as a wildcard to include most of the common expressions that refer to flipped learning (e.g., flipped classroom, flipped instruction, and inverted classroom) or the COVID-19 pandemic. To avoid inadvertently excluding relevant articles, we did not specify subject disciplines (e.g., pathology, pharmacy, and radiology) in the search string. The search was conducted on February 26, 2022.

### Inclusion and exclusion criteria

Empirical studies published between January 2020 and December 2021 (i.e., the first 2 years of the pandemic) were reviewed, including online advance publications. At the time of writing, this period covered all existing research on flipped learning during the pandemic because the first outbreak was reported in December 2019 [19]. Only studies on flipped courses that were conducted and sustained in a fully online environment were included; those that did not fully transfer their face-to-face activities to the online medium were excluded. To ensure consistency, the online flipped courses had to be described clearly and had to satisfy the definition provided by the Flipped Learning Network [9]. Moreover, the courses had to be in the domain of health professions education.

No constraints were imposed on the types of empirical data used (e.g., tests, surveys, and interviews). However, non-empirical studies or articles that provided little empirical evidence were excluded. In addition, no constraints were imposed on the education contexts, the language of instruction, and the location of the studies. However, the manuscripts had to be written in English and published in peer-reviewed journals because peer review is a key criterion for ensuring research quality [20].

### Data extraction and analysis

The following data were extracted from each article: (a) the author(s), the year of publication, and the country of implementation; (b) the course title (or course content, if course title was not available) and student level; (c) the types of pre-class and in-class learning activities; (d) the challenges to practicing online flipped learning (RQ1); and (e) the useful elements related to course design and implementation (RQ2). Although we intended to analyze the effect of online flipped learning on student achievement, only two comparison studies with adequate quantitative data (e.g., the number of students, means, and standard deviation) were found (see Table S1 in Additional file 1), thereby preventing such an analysis.

To answer the research questions, content analyses of the results, findings, discussions, and conclusions of the included articles were conducted [21]. To address RQ1, an initial framework based on the three categories of flipped learning challenges (i.e., student-related, faculty, and operational challenges) defined by Betihavas et al. [10] was adopted for the thematic analysis. To address RQ2, an initial framework based on the RCoI framework (i.e., cognitive, social, teaching, and learner presence), as adopted in Kim et al. [13], was used for the thematic analysis. Although these frameworks provided a basis for the content analysis, we remained open to refining or adding to the frameworks if new categories emerged.

To enhance the consistency of coding, several exemplary quotations (see Tables S2 and S3 in Additional file 1) that clearly illustrated each constructed theme were identified. Multiple reviews of the data were conducted to ensure the correct understanding of each theme [22]. Furthermore, approximately 25% of the articles were double-coded by the second author to establish coding reliability. In the event of disagreement, the authors re-examined the articles in question together, until a consensus was reached.

## Results

### Study selection and characteristics of the included articles

**Fig. 1:**
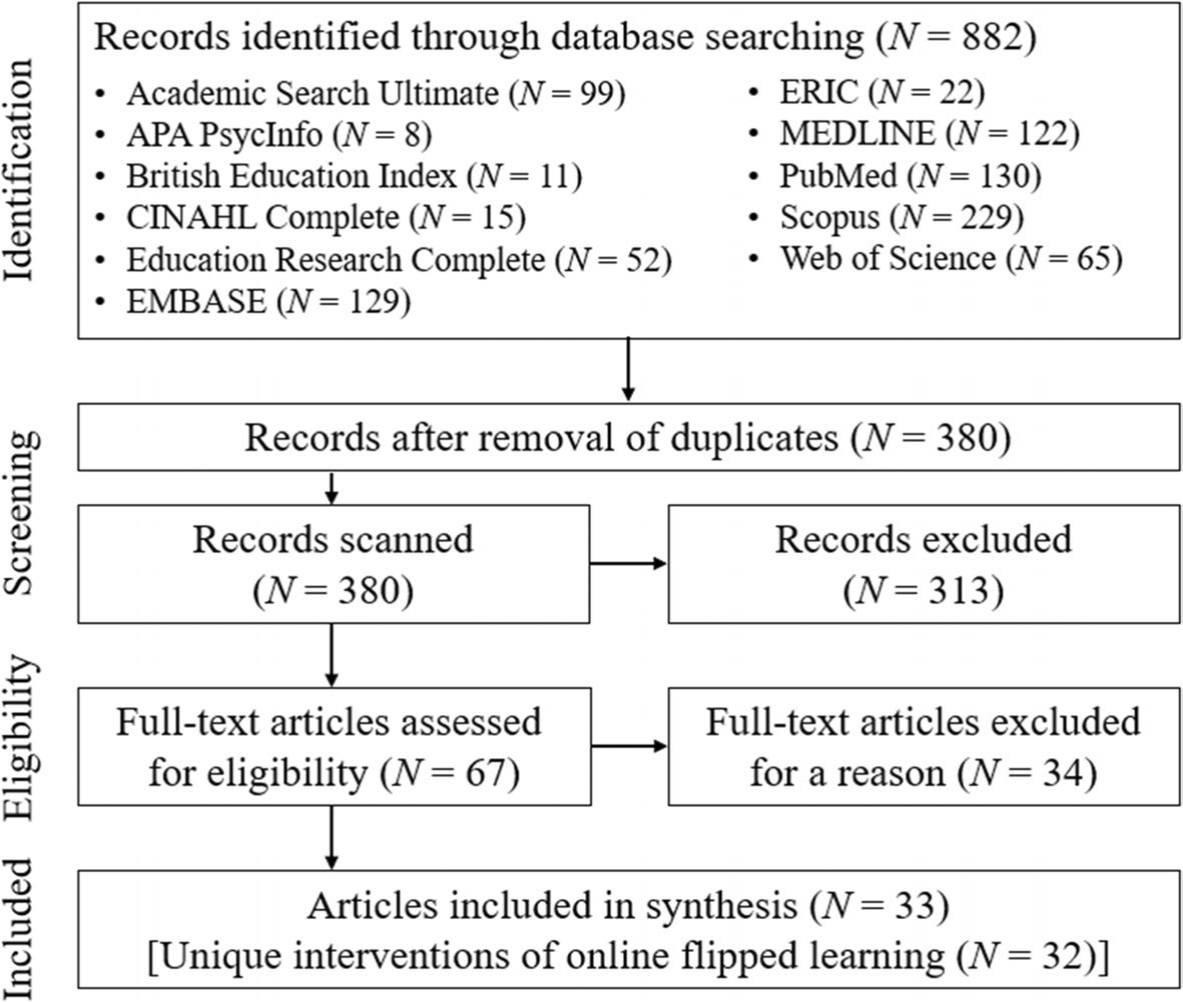
PRISMA flow diagram of article selection procedure

In the database search, 882 peer-reviewed journal articles (published from January 2020 to December 2021) were found by February 26, 2022 (the time of finalizing the search). More than half of these articles were removed due to replication across databases. Although the search string captured a variety of terms referring to flipped learning, it also yielded many irrelevant search outcomes (e.g., viruses of invertebrates) and studies outside of health professions education. Therefore, after reviewing their titles and abstracts, many irrelevant articles (*N* = 313) were excluded. Sixty-seven full-text articles were assessed for eligibility, of which 34 were excluded based on the inclusion and exclusion criteria. The final selection comprised 33 articles. Two of these articles reported on the same intervention by Chaudhuri et al. [23*, 24*], and therefore 32 unique interventions of online flipped learning were analyzed (Table 2). Figure 1 outlines the procedure used for article selection.

**Table 2:** Background summary of the 32 interventions

Study	Location	Course title/content	Grade level
Akram et al. [25*]	Saudi Arabia	Learning skills of medical students	UG
Annamalai et al. [26*]	Malaysia	In-patient pharmacy clerkship	UG
Bartoletta et al. [27*]	The United States	Hand surgery	PG
Carrazoni et al. [28*]	Brazil	Basic concepts in neurophysiology	UG
Chaudhuri et al. [23*, 24*]	India	Physiology	UG
Cho and Kim [29*]	Korea	Nursing and English	UG
DePietro et al. [11*]	The United States	Interventional radiology	UG
Durfee et al. [1*]	The United States	Virtual radiology core clerkship	UG & PG
Gisondo et al. [30*]	The United States	Transport medicine	PG
Gopalan et al. [31*]	The United States	Physiology	PG
Grant et al. [32*]	The United States	Psychiatry	UG
Guiter et al. [33*]	The United States	Pathology	UG
Haftador et al. [3*]	Iran	Nursing	UG
Höhne et al. [34*]	Germany	Ultrasound	UG
Huang et al. [35*]	The United States	Rehabilitation rotation	UG
Khapre et al. [36*]	India	Public health	PG
Kim et al. [37*]	Korea	Radiation oncology	UG
Knie et al. [38*]	Germany	Clinical communication	UG
Lapane and Dube [39*]	The United States	Rigor and reproducibility	PG & PhD
Liu et al. [40*]	China	Medical morphology	UG
Patel and Taggar [41*]	The United Kingdom	Primary care	UG
Perumal-Pillay and Walters [7*]	South Africa	Pharmacy	UG
Qian et al. [42*]	China	COVID-19 knowledge	UG
Rehman and Fatima [43*]	Pakistan	Endocrine reproduction	UG
Roy et al. [44*]	India	Anatomy	UG
Smith and Boscak [45*]	The United States	Trauma and emergency radiology	UG
Soll et al. [46*]	Germany	Cognitive behavior therapy	PG
Teichgräber et al. [8*]	Germany	Clinical radiology	UG
Tiedemann and Simmenroth [47*]	Germany	Alcohol and smoking counselling	UG
Vladis and Coleman [48*]	The United States	Using Python for research	PhD
Wang et al. [2*]	China	Oral histopathology	UG
Xie et al. [49*]	China	Medical molecular biology	UG

**Fig. 2:**
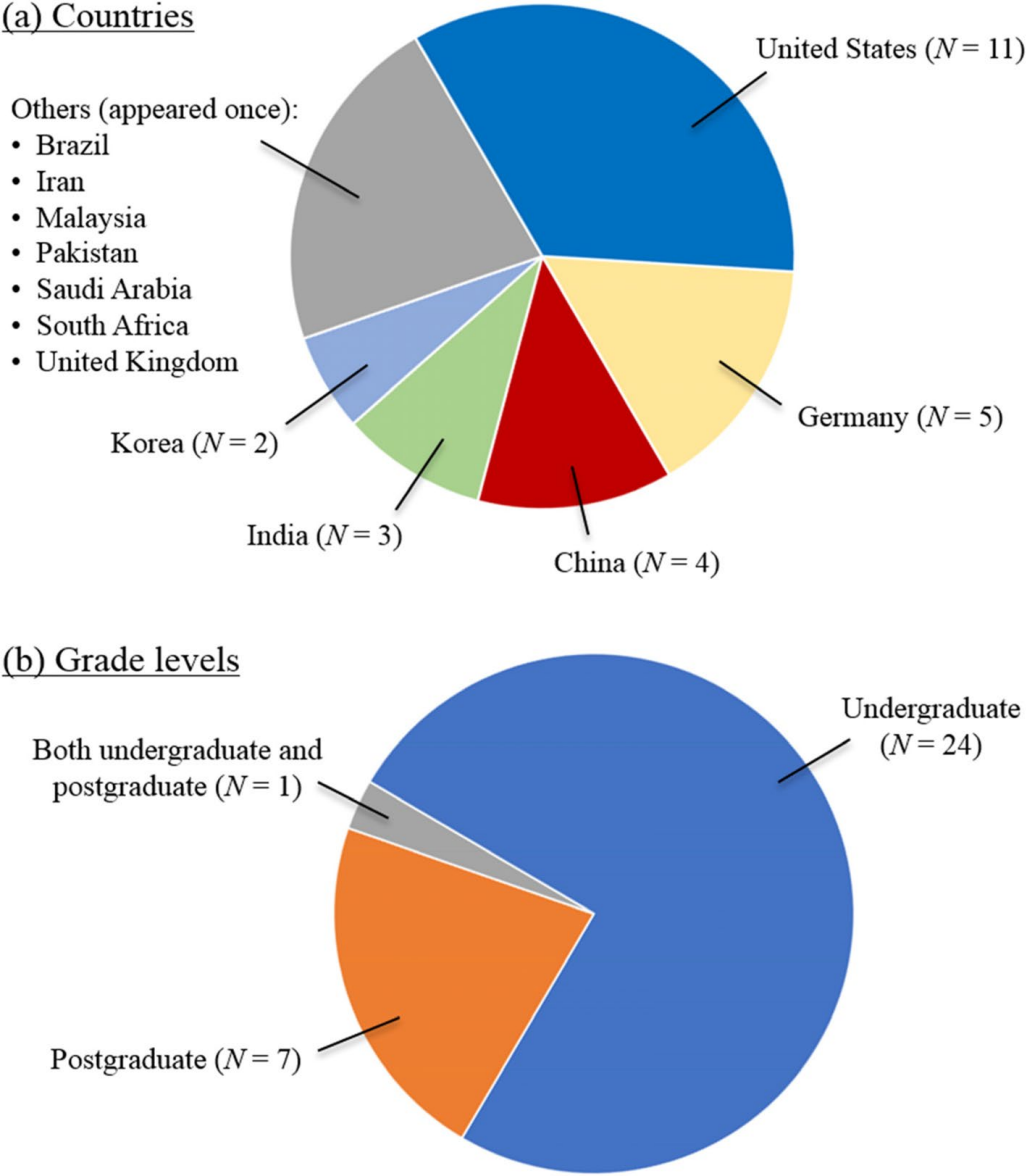
**a** Countries and **b** grade levels of the 32 interventions

Figure 2 illustrates the number of interventions included in this review, and the country in which they were conducted and their grade level. As Fig. 2(a) shows, the majority of the interventions were conducted in the United States (*N* = 11), followed by Germany (*N* = 5), China (*N* = 4), and India (*N* = 3). Figure 2(b) shows that except for the study by Durfee et al. [1*], which involved both undergraduate (UG) and postgraduate (PG) students, the interventions were carried out at the either the UG (*N* = 24) or PG (including PhD; *N* = 7) levels. The courses covered various areas of health professions education, such as radiology [1*, 8*, 11*, 45*], physiology [23*, 24*, 28*, 31*], nursing [3*, 29*], and pharmacy [7*, 26*].

### Instructional activities in online flipped learning

**Fig. 3:**
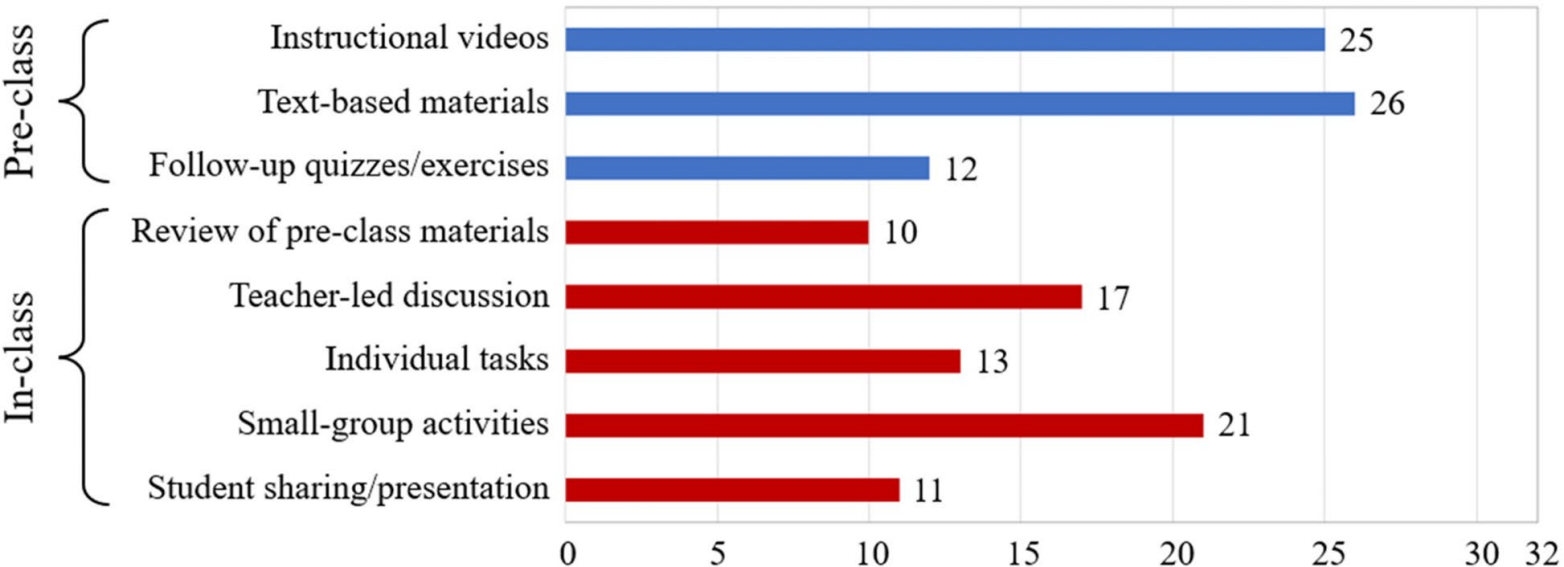
Major pre-class and in-class learning activities in the 32 interventions (the total number of activities is greater than 32 because some courses provided multiple activities)

Figure 3 shows the major pre-class and in-class learning activities adopted in the 32 interventions. Instructional videos (*N* = 25), text-based materials (*N* = 26), and follow-up quizzes or exercises (*N* = 12) were the primary resources used for pre-class learning activities. For example, Haftador et al. [3*] uploaded video clips (i.e., instructional videos) with questions (i.e., follow-up quizzes/exercises) and an electronic book (i.e., text-based materials) for their students. Several studies reported the learning management system that was used to organize pre-class learning activities, such as Blackboard (*N* = 3), Google Classroom (*N* = 3), and Moodle (*N* = 2).

As part of the in-class learning activities, some instructors reviewed (*N* = 10) the pre-class materials at the start of their synchronous online sessions. Teacher-led class discussions (*N* = 17), individual tasks (*N* = 13), small-group activities (*N* = 21), and student sharing or presentation (*N* = 11) were the major activities during classes. For example, in the study by Gopalan et al. [31*], the instructor first encouraged students to ask questions related to the content of the pre-class learning (i.e., review) and then provided mini lectures (i.e., teacher-led discussion). Subsequently, their students completed learning tasks individually and group activities in breakout rooms (i.e., small-group activities). In addition to these major activities, a few instructors distributed in-class assessments at the start of their lessons (e.g., [28*, 29*]) or at regular intervals during classes (e.g., [23*, 24*, 40*]). Finally, we identified the video conferencing platform that was used in 26 of the interventions, which showed that the most frequently used platforms were Zoom (*N* = 16) and Microsoft Teams (*N* = 3).

### RQ1: What are the challenges to online flipped courses during the pandemic?

**Fig. 4:**
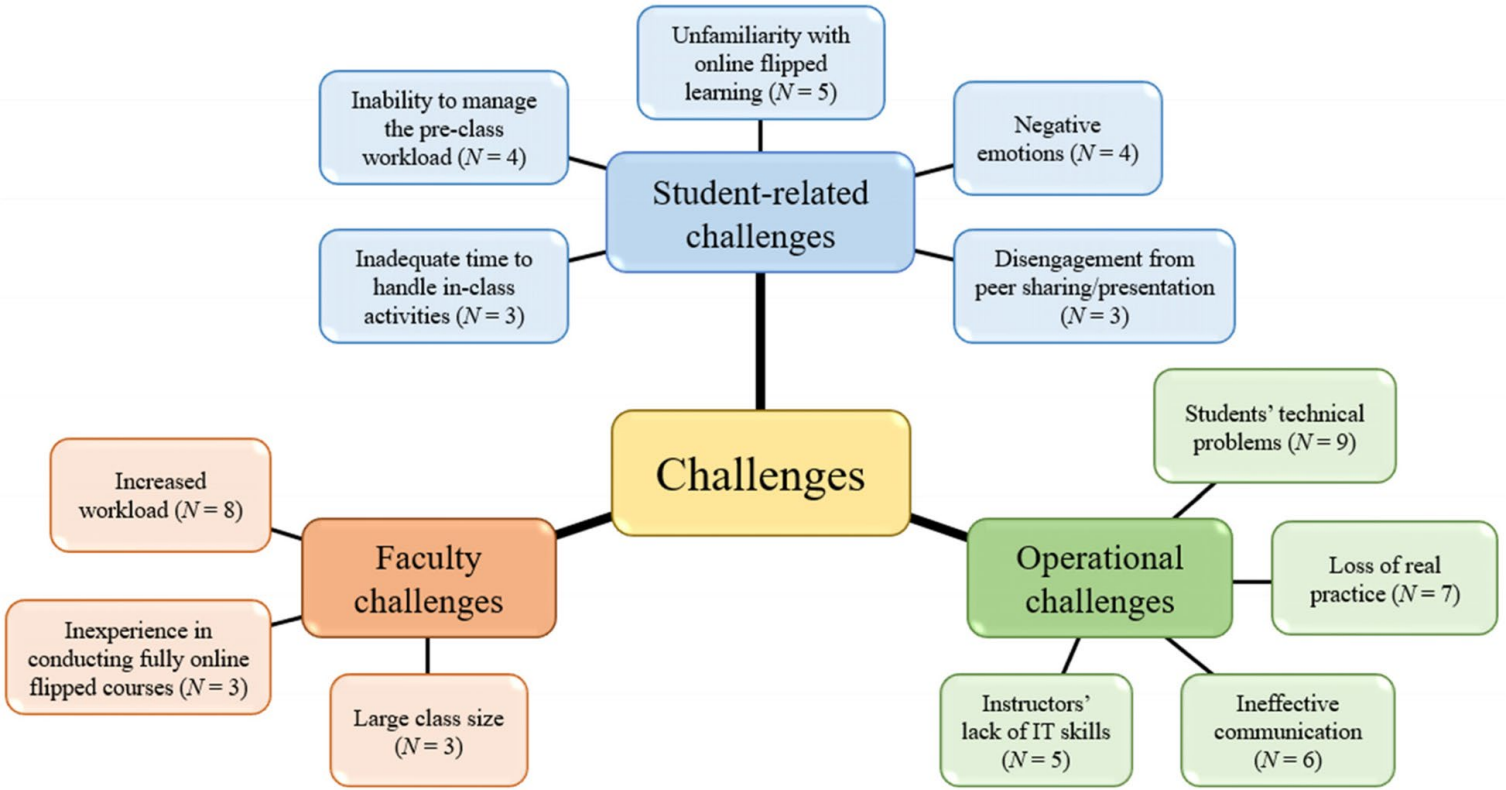
Major challenges to online flipped learning across the 32 interventions

In the reviewed studies, the researchers reported various challenges to the practice of online flipped learning during the pandemic. Based on the framework of Betihavas et al. [10], we identified and organized the major challenges into the three main themes of student-related challenges, faculty challenges, and operational challenges across the 32 interventions. As Fig. 4 and Table S2 in Additional file 1 show, these themes were further categorized into sub-themes.

First, five major student-related challenges to online flipped learning were identified, namely, unfamiliarity with online flipped learning (*N* = 5), inability to manage the pre-class workload (*N* = 4), negative emotions (*N* = 4), inadequate time to handle in-class activities (*N* = 3), and disengagement from peer sharing/presentation (*N* = 3). For example, an inability to manage the pre-class workload was mentioned in the study by Akram et al. [25*], in which some students stated that they did not have sufficient time prior to the class to view the pre-class videos.

Second, three major faculty challenges to online flipped learning were identified: increased workload (*N* = 8), inexperience in conducting fully online flipped courses (*N* = 3), and large class size (*N* = 3). Among these, increased workload was most frequently mentioned. In the words of one instructor of Teichgräber et al. [8*], “Preparation of new learning videos needs initial additional effort” (p. 8).

Third, several studies mentioned operational challenges, and these were in fact the most frequently mentioned challenges. This theme was classified into four sub-themes: students’ technical problems (*N* = 9), loss of real practice (*N* = 7), ineffective communication (*N* = 6), and instructors’ lack of IT skills (*N* = 5). An example of the loss of real practice was found in Kim et al. [37*], who mentioned that their students “did not have the opportunity to experience outpatient follow-up or toxicity management after radiation therapy, [which] could be a major limitation for them” (p. 8).

### RQ2: What are the useful elements of the design and implementation of online flipped courses?

**Fig. 5:**
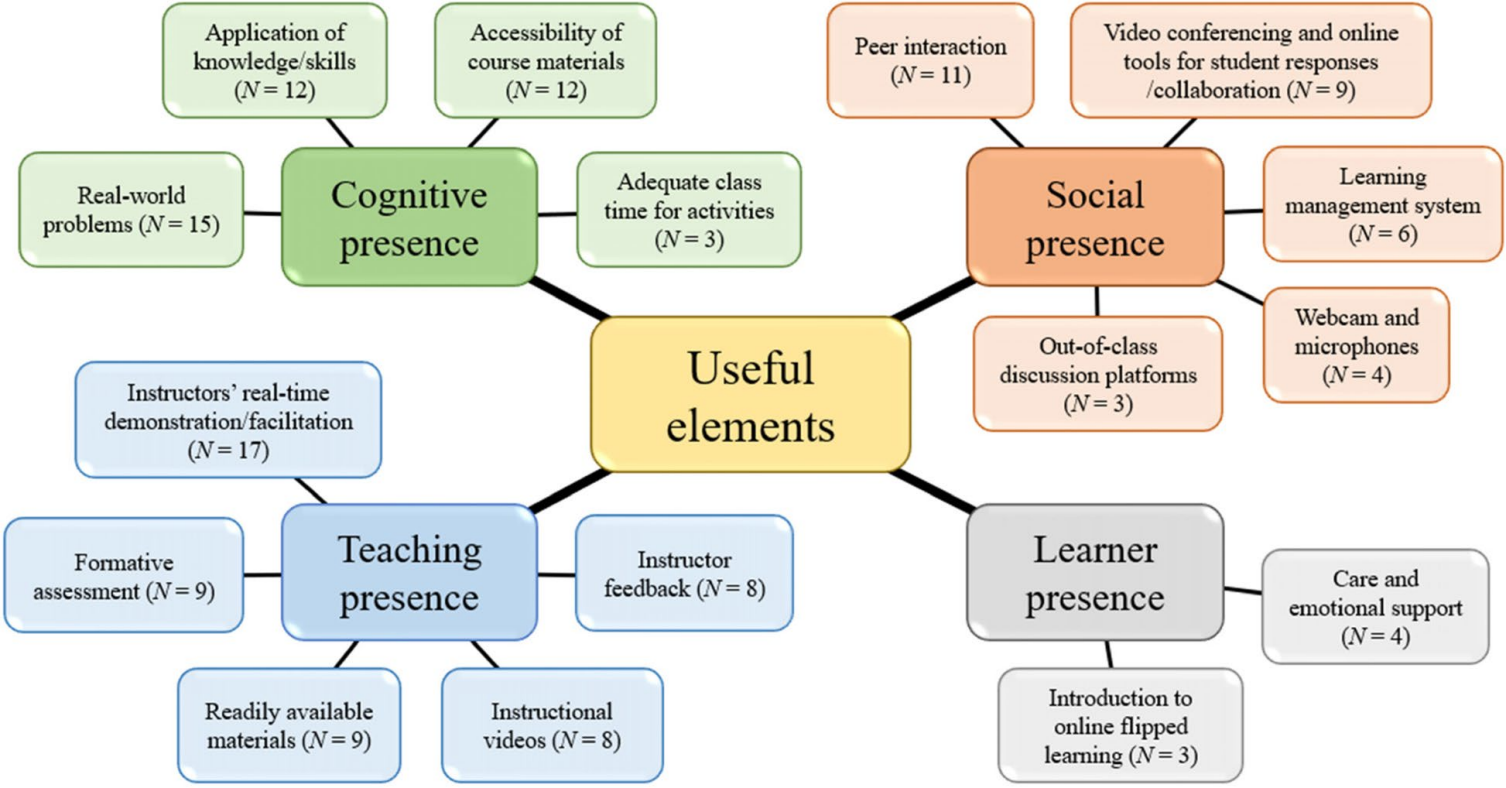
Major useful course-design elements for online flipped learning across the 32 interventions

We identified several elements that instructors and students found useful in designing and implementing online flipped courses. These elements were organized based on the RCoI framework [13, 17]. Figure 5 and Table S3 in the Additional file 1 summarize the useful elements across the four major themes of the framework: cognitive presence, social presence, teaching presence, and learner presence.

First, the useful elements related to cognitive presence were classified into four sub-themes: real-world problems (*N* = 15), application of knowledge/skills (*N* = 12), accessibility of course materials (*N* = 12), and adequate class time for activities (*N* = 3). Of these, the use of real-world problems was most frequently mentioned, with respect to which Perumal-Pillay and Walters [7*] noted that “Case-based learning used human cases to link theory to practice and allowed for simulation of the actual working environment, which was especially useful during students’ role-play demonstrations” (p. 169).

Second, the useful elements related to social presence were classified into five sub-themes: peer interaction (*N* = 11), video conferencing and online tools for student responses/collaboration (*N* = 9), learning management system (*N* = 6), webcam and microphones (*N* = 4), and out-of-class discussion platforms (*N* = 3). For example, Perumal-Pillay and Walters [7*] highlighted the importance of a video conferencing platform and learning management system for sustaining group activities in a fully online environment. In their words, “Moodle and Zoom breakout rooms allowed the toolbox to be successfully applied to numerous synchronous online pharmacy skills group-work sessions” (p. 169).

Third, the useful elements related to teaching presencewere classified into five sub-themes: instructor’s real-time demonstration/facilitation (*N* = 17), formative assessment (*N* = 9), readily available materials (*N* = 9), instructional videos (*N* = 8), and instructor feedback (*N* = 8). Of these, instructor’s real-time demonstration/facilitation was the most frequently mentioned. In the opinion of Perumal-Pillay and Walters [7*], “facilitators should visit each breakout room every 10 min to moderate discussions and pose questions to encourage participation” (p. 168).

Finally, there were two sub-themes related to learner presence: care and emotional support (*N* = 4) and introduction to online flipped learning (*N* = 3). For example, Chaudhuri et al. [24*] touched upon care and emotional support, as when they reported that “Many of our students complained that they were feeling very stressed” (p. 64). They thus emphasized the importance of assessing students’ stress levels and using the support of the stress management clinic in their university.

## Discussion

During the first 2 years of the COVID-19 pandemic, instructors across various parts of the world maintained the continuity and quality of health professions education by adopting flipped learning in a fully online environment. The findings of this review suggest that the major pre-class learning activities (i.e., instructional videos, text-based materials, and follow-up quizzes/exercises) and in-class learning activities (i.e., teacher-led discussion, individual tasks, and small-group activities) that they adopted in flipped courses were generally similar to those used before the pandemic (for a review, see [6, 10, 21]). Despite these similarities, the interactive classrooms during the pandemic were held via a video conferencing platform such as Zoom or Microsoft Teams.

However, some challenges emerged when instructors designed and implemented their online flipped courses (see Fig. 4 and Table S2 in Additional file 1), including student-related challenges (e.g., unfamiliarity with online flipped learning and negative emotions), faculty challenges (e.g., increased workload), and operational challenges (e.g., ineffective communication). To address these challenges and to improve future practice, we first identified various useful course-design elements for online flipped learning across studies (see Fig. 5 and Table S3 in Additional file 1). Next, we used the RCoI framework [13, 17] to help reflect on these findings. Based on this theoretical framework, we derived a set of nine principles for the effective practice of this instructional approach, as shown in Table 3. By leveraging the identified useful course-design elements, these principles can facilitate different types of presence of the RCoI framework and can address the major challenges to online flipped learning.

**Table 3:** Principles of effective online flipped learning practice by type of presence

Principles	Supporting elements (challenges addressed)
Cognitive presence	
• Engage students in real-world problems and applications of knowledge and skills (Principle 7)	Real-world problems; application of knowledge/skills
• Allow adequate time for students to complete pre-class and in-class activities (Principle 2)	Accessibility of course materials (SC: Inability to manage the pre-class workload); adequate class time for activities (SC: Inadequate time to handle in-class activities)
Social presence	
• Organize small-group activities to facilitate peer interaction (Principle 8)	Peer interaction
• Use a learning management system and discussion platform that facilitate pre-class learning (Principle 3)	Learning management system; out-of-class discussion platforms
• Use online interactive tools that facilitate synchronous learning activities (Principle 5)	Video conferencing and online tools for student responses/collaboration; webcam and microphones
Teaching presence	
• Use instructional videos to support students’ pre-class learning (Principle 4)	Instructional videos; readily available materials (FC: Increased workload)
• Allocate class time for instructor demonstration and facilitation (Principle 6)	Instructor’s real-time demonstration/facilitation
• Provide formative assessment with instructor feedback (Principle 9)	Formative assessment; instructor feedback
Learner presence	
• Manage the transition to online flipped learning with emotional support (Principle 1)	Introduction to online flipped learning (SC: Unfamiliarity with online flipped learning); care and emotional support (SC: Negative emotions)

*SC* Student-related challenges, *FC* Faculty challenges, *OC* Operational challenges

For ease of reference, the principles are organized and presented in the following sequence: one principle that is applicable at the very beginning of the flipped courses is discussed first, followed by those that pertain to pre-class learning and then those that pertain to in-class learning.

However, it is acknowledged that several identified challenges (e.g., the operational challenge of loss of real practice) cannot be addressed based on the findings of this review. Therefore, we discuss some initial solutions in the “Limitations and recommendations for future research” section.

### Principle 1: Manage the transition to online flipped learning with emotional support

Although flipped learning existed before the pandemic, some students may not be familiar with its application in a fully online environment [31*, 44*]. Therefore, instructors should orient students to their online flipped courses [8*]. For example, Perumal-Pillay and Walters [7*] noted that some students required an introduction to the concept of breakout rooms and the format for online group work. Furthermore, care and emotional support are essential to address negative emotions of students such as dissatisfaction [26*], stress, and anxiety [24*, 49*]. These negative emotions might be due to the pandemic or their study. Instructors can assess students’ mental health and follow up on their status regularly [24*]. Those students who experience serious emotional disturbances should be referred to counselors and psychological interventions as necessary [49*].

### Principle 2: Allow adequate time for students to complete pre-class and in-class activities

The findings of this review highlight the importance of allowing adequate time for students to complete pre-class and in-class activities. In Akram et al. [25*], for example, some students reported that they did not have sufficient time to prepare for their classes because their family members were infected with COVID-19. Therefore, instructors should make their course materials accessible online earlier than has been the case, to allow enough time for students to deal with unexpected problems. In a survey of nearly 100 students conducted by Rehman and Fatima [43*], approximately 90% of the students expressed that a 1-week window was sufficient for managing their pre-class workload. Furthermore, instructors should be aware that students may need more time to complete certain in-class activities in a fully online environment than in a classroom environment [31*, 39*, 47*]. These activities include group discussions [8*], group work [39*], and role-playing [47*].

### Principle 3: Use a learning management system and discussion platform that facilitate pre-class learning

Appropriate technology tools can facilitate students’ pre-class learning. First, a learning management system (e.g., Blackboard, Google Classroom, and Moodle) can help instructors organize their course materials. For example, Liu et al. [40*] uploaded all of their learning resources (e.g., videos, materials, chapter tests, homework, and case analysis/discussion) to their system, which enabled their students to access and prepare for their class anytime and anywhere. Second, an online discussion forum is key to supporting effective teaching and learning [43*], and it allows students to seek help from their instructor and peers while preparing for a class. Cho and Kim [29*] observed that the online discussion forum hosted a large number of student–student and student–instructor interactions during their online flipped course.

### Principle 4: Use instructional videos to support students’ pre-class learning

Students in multiple studies (e.g., [8*, 39*, 47*]) responded favorably to instructional videos. For example, the students in a study by Gisondo et al. [30*] expressed that videos were extremely effective for teaching the basic principles and safety aspects of transport medicine. Students could revisit video lectures multiple times to gain a better understanding of the content [25*, 31*, 43*]. However, the production of learning resources increases instructor workload. To alleviate this, Durfee et al. [1*] converted the teaching resources from their traditional radiology course into a fully online format, whereas Teichgräber et al. [8*] suggested using external online resources. Echoing the suggestion of Teichgräber et al. [8*], Vladis and Coleman [48*] used the high-quality videos available on HarvardX (free online courses from Harvard University) and thus expended minimal effort on course development.

### Principle 5: Use online interactive tools to facilitate synchronous learning activities

To compensate for the lack of a physical classroom, appropriate technology tools that facilitate students’ in-class learning should be used. In the reviewed studies, some instructors mentioned how the features of their video conferencing and online tools supported their delivery of class activities. For example, Teichgräber et al. [8*] reported that the use of a student response system stimulated the plenary debate on clinical cases. Similarly, Lapane and Dube [39*] used the polling option in Zoom for students to vote after debates. They further used the breakout rooms feature in Zoom to create virtual rooms for interactive group activities. To address the challenge of ineffective communication reported in some studies (e.g., [29*, 46*]), instructors can encourage students to use a webcam and microphone to facilitate interactions. For example, Patel and Taggar [41*] recommended that their students kept their camera and microphone on to facilitate interactions during small-group activities and consultations.

### Principle 6: Allocate class time for instructor demonstration and facilitation

Despite the popularity of instructional videos, in-class demonstration and facilitation by instructors remain essential in online flipped courses. Multiple studies found that students valued the interactions with their instructors during synchronous online sessions, which enhanced their interest [46*], satisfaction [29*], and critical thinking [26*]. Through real-time interactions, instructors can deepen students’ understanding of pre-class materials [40*], clear their misconceptions [23*], explain complex concepts [31*], and guide them to analyze novel cases [45*]. Furthermore, students may not be able to effectively acquire certain knowledge and skills in the health professions, especially those involving interventions with clients or patients, from instructional videos and text-based materials. For example, Soll et al. [46*] shared that their students were often interested in their instructor performing a therapeutic intervention role-play, with a student playing the role of a client. The researchers stated that this real-time active learning experience facilitated the subsequent training of students in performing interventions.

### Principle 7: Engage students in real-world problems and application of knowledge and skills

As the students in the study by Lapane and Dube [39*] expressed, the content of a course should be practical and relevant to students’ possible future careers and professional life situations. Therefore, Carrazoni et al. [28*] emphasized the importance of incorporating clinically relevant topics into a course. Case-based learning based on human and clinical cases can be adopted to link theory to practice and simulate the actual working environment [7*, 42]. Furthermore, Annamalai et al. [26*] provided students with opportunities to experience the actual role of a pharmacist in their online flipped in-patient clerkship, which helped them to “imagine working in the hospital” (p. 6). These activities engage students in active learning through the application of knowledge and skills to solve problems, and thus lead to positive learning outcomes [26*, 42, 43*].

### Principle 8: Organize small-group activities to facilitate peer interaction

Students in several of the reviewed studies (e.g., [31*, 33*, 39*]) appreciated opportunities to learn from peers in small-group activities. Perumal-Pillay and Walters [7*] noted that group work is essential for improving students’ professional communication skills. In this review, we found three strategies to facilitate and sustain group dynamics in virtual discussion rooms. First, Teichgräber et al. [8*] recommended self-chosen grouping when forming groups, as they observed that students’ motivation and cooperation were low if random grouping was used. Second, Grant et al. [32*] and Patel and Taggar [41*] suggested keeping group membership consistent to help students build their group identity and increase their engagement. Finally, instructors can consider using ability grouping (i.e., grouping students of similar abilities), which would allow them to tailor their support for each group [48*].

### Principle 9: Provide formative assessment with instructor feedback

As in conventional flipped courses [13], formative assessment and instructor feedback are important to facilitate student learning in fully online flipped courses. In the reviewed studies, some instructors applied pre-class assignments via Google Form [26*], in-class problem-solving tasks (e.g., [31*, 48*]), and weekly assignments (e.g., [45*]) as their formative assessments. In the words of one instructor of Lapane and Dube [39*], these assessments were “helpful to instructors to know what students absorbed” (p. 1032). Instructors can provide students with feedback based on their performance, correct their mistakes [8*, 31*, 45*], and enhance their mastery of course materials [40*, 41*].

### Limitations and recommendations for future research

Several limitations in this review must be acknowledged. First, the coding and analysis conducted during the review could only be based on what had been reported in the included articles. The absence of certain themes in the coding implies that these had not been reported explicitly in the articles. There may thus be discrepancies between what was documented in the articles and actual practice. For example, whereas Kim et al. [13] emphasized the importance of providing students with incentives for class preparation, the reviewed studies lacked an explicit discussion on the necessity of this element. Second, the majority of the reviewed studies were conducted at the undergraduate level and in the United States. The findings of this review may thus be biased toward these specific contexts. Further studies in other education contexts are necessary. Third, we found only a few studies that compared the quantitative effects of online flipped learning with those of other instructional approaches, such as online traditional lecturing. Further research is necessary to compare such instructional approaches based on objective measures of student achievement (e.g., examinations and post-tests).

Finally, follow-up studies are required to validate and enhance the developed principles. Research on how to support online flipped learning practice at the institutional level must also be conducted. As Chaudhuri et al. [23*] lamented, “Our institution and university could not provide us with any support and guideline” (p. 611). Several challenges that were identified in this review must be addressed beyond the course level. The following are some examples.

- The faculty challenge of inexperience in conducting fully online flipped courses: institutions could address this by providing professional development opportunities for instructors.
- The faculty challenge of large class size: institutions could allocate additional instructors to reduce the size of each class.
- The operational challenge of students’ technical problems: institutions could provide students in need with sufficient hardware and Internet data to sustain their participation in online flipped courses.
- The operational challenge of loss of real practice: Kim et al. [37*] suggested developing new digital and virtual reality (VR) systems to help substitute for real-world practice. This technology can create a simulated environment where students can interact with three-dimensional objects as in the real world [50].

## Conclusion

In this review, we examined the research available on the practice of online flipped learning in health professions education during the COVID-19 pandemic. Thirty-three empirical studies were analyzed. Although a number of student-related, faculty, and operational challenges to online flipped learning were found, we identified various useful elements related to course design and implementation. Based on these findings and the RCoI framework, this review contributes to the literature by developing nine principles for effective online flipped learning practice. These principles were established on the basis of empirical evidence and aimed at addressing the major challenges identified in the reviewed studies. Thus, these principles provide insights for health professions instructors intending to offer rigorously designed online flipped courses.

Nevertheless, the findings of this review and the developed principles may be biased toward a specific context (i.e., the United States and the undergraduate level) and limited by what was documented in the included articles. Further research is required to validate and enhance these principles across multiple education contexts. 

## Supplementary Information

**Additional file 1: Table S1.** Quantitative results of the comparison studies that applied online flipped learning in their experimental group. **Table S2.** Major challenges to online flipped learning across the 32 interventions by count. **Table S3.** Major useful course-design elements of online flipped learning across the 32 interventions by count.

## Data Availability

The datasets used and/or analyzed during the current study available from the corresponding author on reasonable request.
