# Supplementary material for: Design principles for fully online flipped learning in health professions education: a systematic review of research during the COVID-19 pandemic
Source: BMC Med Educ. 2022 Oct 13;22:720. doi: 10.1186/s12909-022-03782-0 (PMC9559249; doi:10.1186/s12909-022-03782-0)
Supplement: Supplementary file 1 — Additional file 1: Table S1. Quantitative results of the comparison studies that applied online flipped learning in their experimental group. Table S2. Major challenges to online flipped learning across the 32 interventions by count. Table S3. Major useful course-design elements of online flipped learning across the 32 interventions by count. [file 12909_2022_3782_MOESM1_ESM.docx]

**Additional file 1**

**Table S1.** Quantitative results of the comparison studies that applied online flipped learning in their experimental group.

|  | Online flipped learning | | Comparison group | |  |  |
| --- | --- | --- | --- | --- | --- | --- |
| Study | *n* | *M* (*SD*) | *n* | *M* (*SD*) | *p* | *d* ^{1}^ |
| Compared to face-to-face flipped learning | | | | | | |
| - Teichgräber et al. [8]: examination grade **^{2}^** | **266** | **1.4 (0.6)** | 266 | 1.8 (0.7) | **< .001** | **.61** |
| Compared to online traditional lecturing | | | | | | |
| - Qian et al. [42]: post-test | **37** | **86.22 (5.74)** | 37 | 79.78 (7.12) | **< .001** | **1.00** |
| - Qian et al. [42]: retention test (2 months later) | **37** | **76.03 (6.49)** | 37 | 67.16 (5.05) | **< .001** | **1.53** |

Bold values indicate significant results.

*Note:*

{1} The effect sizes were calculated using the following formulas

$$\text{Cohe}\text{n}'\text{s }d=\frac{M_{OFL}-M_{CG}}{SD_{pooled}}$$

where OFL = Online flipped learning, CG = Comparison group, and

$SD_{pooled}=\sqrt{\frac{SD_{OFL}^{2}+SD_{CG}^{2}}{2}}$.

{2} The assessment scores ranged from 1 (excellent) to 5 (inadequate).

**Table S2.** Major challenges to online flipped learning across the 32 interventions by count.

| Themes and sub-themes | Count | Representative citations |
| --- | --- | --- |
| Student-related challenges |  |  |
| - Unfamiliarity with online flipped learning | 5 | Annamalai et al. [26]: “Students are still in their adaption phase for online clerkship and it is not surprising for such reluctance” (p. 9). |
| - Inability to manage the pre-class workload | 4 | Akram et al. [25]: “Some respondents [students] agreed that the time they were given to watch the video was too short, and they could thus not watch the video properly” (p. 37). |
| - Negative emotions | 4 | Xie et al. [49]: “The results showed that most students felt slight or medium stressed and anxious about the coronavirus (COVID-19) outbreak, and a small number of students may have experienced serious emotional disturbance” (p. 724). |
| - Inadequate time to handle in-class activities | 3 | Gopalan et al. [31]: “some students struggled to log in quickly and a few students required more time to complete the assessment” (p. 676). |
| - Disengagement from peer sharing/presentation | 3 | Liu et al. [40]: “There is a lack of school atmosphere; thus, students lacked interest in peer feedback” (p. 15). |
| Faculty challenges |  |  |
| - Increased workload | 8 | Teichgräber et al. [8]: “Preparation of new learning videos needs initial additional effort” (p. 8). |
| - Inexperience in conducting fully online flipped courses | 3 | Grant et al. [32]: “the FC [flipped classroom] approach was a new experience for many facilitators… the abrupt transition to virtual instruction, with facilitators unfamiliar with the methodology, exacerbated perceived challenges with student engagement” (p. 1139). |
| - Large class size | 3 | Durfee et al. [1]: “Creating an interactive learning environment in our virtual radiology course, however, was particularly challenging given the large class size” (p. 1465). |
| Operational challenges |  |  |
| - Students’ technical problems | 9 | Chaudhuri et al. [23]: “Many of our students are from remote villages having poor network connectivity. Hence, it is very difficult for them to access online classes through different Apps. Most of them do not have laptops or desktops. They depend mostly on mobile phones to continue their learning online” (p. 610). |
| - Loss of real practice | 7 | Kim et al. [37]: “the fact that the students did not have the opportunity to experience outpatient follow-up or toxicity management after radiation therapy, could be a major limitation for them” (p. 8). |
| - Ineffective communication | 6 | Soll et al. [46]: “emerging discussions in web conferences are hindered by the fact that social cues such as eye gaze and body gestures cannot be used in an online setting to determine the audience’s degree of interest and adapt speech content towards it” (p. 6). |
| - Instructors’ lack of IT skills | 5 | Akram et al. [25]: “When the flipped method was adopted, many teachers had difficulty proceeding with their teaching because they were not experts in technology use” (p. 38). |

**Table S3.** Major useful course-design elements of online flipped learning across the 32 interventions by count.

| Themes and sub-themes | Count | Representative citations |
| --- | --- | --- |
| Cognitive presence |  |  |
| - Real-world problems | 15 | Perumal-Pillay and Walters [7]: “Case-based learning used human cases to link theory to practice and allowed for simulation of the actual working environment, which was especially useful during students’ role-play demonstrations” (p. 169). |
| - Application of knowledge/skills | 12 | Annamalai et al. [26]: “The activities designed by the lecturer for this online clerkship involved a case-based study that requires critical thinking and problem solving skills that were related to active learning. All these aspects have led to positive learning outcomes” (p. 8). |
| - Accessibility of course materials | 12 | Roy et al. [44]: “Total 92% of the students favoured the sharing of study material at least well in advance days before the class session” (p. 2). |
| - Adequate class time for activities | 3 | Lapane and Dube [39]: Student: “Discussion time was sometimes too short; devote more time to small group work” (p. 1032). |
| Social presence |  |  |
| - Peer interaction | 11 | Gopalan et al. [31]: Students: “Discussion with my peers clarify content I was struggling with” and “Great way to learn from each other and explain topics” (p. 677). |
| - Video conferencing and online tools for student responses/collaboration | 9 | Perumal-Pillay and Walters [7]: “… Zoom breakout rooms allowed the toolbox to be successfully applied to numerous synchronous online pharmacy skills group-work sessions” (p. 169). |
| - Learning management system | 6 | Akram et al. [25]: “The implementation of a viable and effective online learning platform and the use of digitally enabled resources managed properly to provide a scope of other positive results…” (p. 39). |
| - Webcam and microphones | 4 | Patel and Taggar [41]: “During the consultation skills mini-surgeries only the consulting student and patient actors were allowed to keep their microphone and video camera on – this was to enable a greater fidelity of a 1:1 virtual consultation as would be encountered within clinical practice” (p. 297). |
| - Out-of-class discussion platforms | 3 | Cho and Kim [29]: “Both groups had the online bulletin board for mutual communication… In the case of non-face-to-face flipped learning, there was a high degree of individual interactions” (p. 10). |
| Teaching presence |  |  |
| - Instructor’s real-time demonstration/facilitation | 17 | Liu et al. [40]: “Through screen-to-screen experiment teaching, the classroom teaching content is consolidated and the theoretical course understanding is deepened” (p. 14). |
| - Formative assessment | 9 | Smith and Boscak [45]: “The weekly Pacsbin assessments were untimed and not formally graded, serving as formative assessments, a ‘low-stakes’ method intended to stimulate learning and provide feedback” (p. 450). |
| - Readily available materials | 9 | Durfee et al. [1]: “The readily accessible teaching material was helpful for faculty recruitment. Little preparation was necessary to teach” (p. 1462). |
| - Instructional videos | 8 | Akram et al. [25]: Student: “I think the videos help a lot. Watching videos about the information, like simulation, will [help you] remember the information” (p. 37). |
| - Instructor feedback | 8 | Patel and Taggar [41]: Student: “He [the instructor] was excellent with constructive feedback and providing learning/development points for all the students” (p. 300). |
| Learner presence |  |  |
| - Care and emotional support | 4 | Xie et al. [49]: “the psychological issues which accompany this pandemic have rapidly compounded its public health burden… It is particularly important to carry out necessary psychological intervention on college students” (p. 727). |
| - Introduction to online flipped learning | 3 | Perumal-Pillay and Walters [7]: “For students. They required orientation to the concept of breakout rooms at the outset and during the first two sessions they could familiarise themselves with this format for online group work” (p. 168). |
